# Workers with Cardiac AIMD Exposed to EMF: Methods and Case Studies for Risk Analysis in the Framework of the European Regulations

**DOI:** 10.3390/ijerph18189709

**Published:** 2021-09-15

**Authors:** Eugenio Mattei, Federica Censi, Giovanni Calcagnini, Rosaria Falsaperla

**Affiliations:** 1 Department of Cardiovascular, Endocrine-Metabolic Diseases and Aging, National Institute of Health, 00199 Rome, Italy; federica.censi@iss.it (F.C.); giovanni.calcagnini@iss.it (G.C.); 2 Department of Occupational and Environmental Medicine, Epidemiology and Hygiene, Italian Workers’ Compensation Authority, 00078 Roma, Italy; r.falsaperla@inail.it

**Keywords:** occupational safety, electromagnetic field, pacemaker, implantable cardioverter defibrillator

## Abstract

Workers with cardiac active implantable medical devices (AIMD), such as a pacemaker (PM) or an implantable defibrillator (ICD), are considered by the occupational health and safety regulation framework as a particularly sensitive risk group that must be protected against the dangers caused by the interference of electromagnetic field (EMF). In this paper, we first describe the general methodology that shall be followed for the risk assessment of employees with a cardiac AIMD exposed to EMF, according to the EU regulation, and in particular to the EN 50527-2-1:2016 and 50527-2-2:2018 standards. Then, three case studies related to specific EMF sources are presented, to better describe how the initial analysis of the risk assessment can be performed in practice, and to understand if a further specific risk assessment analysis is required or not.

## 1. Introduction

Employers have duties under health and safety laws to assess risks in the workplace. Risk assessment should identify all risks that might cause harm in the workplace and should put in place protective or preventive measures to reduce the risks identified. Within the European Union, the general arrangements for ensuring the health and safety of workers are set out in the Framework Directive (89/391/EEC) [1]. The Electromagnetic Fields (EMF) Directive (2013/35/EU) [2] specifically address the “minimum health and safety requirements regarding the exposure of workers to the risks arising from physical agents (electromagnetic fields)”. According to the EMF Directive, risks to workers may result from both direct effects of the field on the body, and indirect effects, which result from the presence of objects in the field. The indirect effects include the interference with active implanted medical devices, such as cardiac pacemakers (PM) or implantable defibrillators (ICD). This is why workers bearing these types of devices are a group considered to be at particular risk from EMF. Indeed, these workers may not be adequately protected by the action levels specified in the EMF Directive and so it is necessary for employers to consider their exposure separately to that of other workers. PM, ICD and, more in general, active implantable medical devices (AIMD), are known to be susceptible to strong EMF. The MAUDE-database of the American Food and Drug Administration [3]—a database which houses medical device reports submitted by mandatory reporters (manufacturers, importers and device user facilities) and voluntary reporters such as health care professionals, patients and consumers—reveals for the last 5 years 553 cases of malfunctions of PM or ICD due to “electromagnetic interference” (EMI) or “Electromagnetic Compatibility Problem”. Even if, in most cases, the data provided by the MAUDE-database do not allow one to clearly identify the EMF source that caused the EMI, they still represent valuable information that contributes to benefit-risk assessments of the products available on the market. In recent years, AIMD implants have increased exponentially, reaching an annual implantation rate above 260,000 in Italy, Germany, France, and the UK [4,5]. In parallel to expanded cardiac AIMD utilization, exposure to exogenous EMFs from sources such as high-voltage power lines, electronic article surveillance (EAS) systems or electrical appliances both in daily life and in the work environment has similarly increased. The combination of these aspects justifies the great emphasis that the EMF directive gives to the risk analysis of workers bearing cardiac AIMD. Indeed, it is explicitly stressed that: “a system ensuring a high level of protection as regards the adverse health effects and safety risks that may result from exposure to EMF should take due account of specific groups of workers at particular risk and avoid interference problems with, or effects on the functioning of, medical devices such as PM and ICD”. The risk assessment procedure that, according to the EU regulation, shall be followed to guarantee the safety of employees with a cardiac AIMD exposed to EMF is not always straightforward. Indeed, it involves different areas of expertise (e.g., occupational health and safety experts, occupational physician, AIMD-employee’s responsible physician, manufacturer of the AIMD), which are not trivial, in particular for small or medium business companies. Thus, guidelines and documents that can support the employer during the risk assessment evaluation are very important. In this paper, we first explore the general methodology described in the technical standards EN 50527-2-1:2016 and EN 50527-2-2:2018, which specifically addresses the procedures for the assessment of the exposure to EMFs of workers bearing PM and ICD, respectively. Then, three case studies related to specific EMF sources are presented and discussed. In particular, the EMF sources taken as examples are:

1. RFID;
2. Wi-Fi and Bluetooth;
3. UMTS and LTE.

## 2. Materials and Methods

### General Procedure for the Risk Assessment Required for an AIMD Employee

The risk assessment starts from the knowledge of the electromagnetic immunity requirements that AIMD shall comply with before entering the market. In particular, the new European Medical Device Regulation (MDR) [6] recognizes that electromagnetic immunity is an essential requirement for both non-implantable and implantable medical devices. Conformity to the requirements of the MDR can be demonstrated by applying the harmonized standards specific for each particular medical device [7]. The harmonized standards are not mandatory, but contain technical information on the test and the procedures that manufacturers can follow to obtain the presumption of conformity to the requirements of the MDR. The general standard that applies to an AIMD is the EN 45502-1 [8], together with all of the particular standards, specific for the different types of devices (EN 45502-2-1 [9] for the PM, EN45502-2-2 for ICD [10], etc.). The immunity levels adopted in these standards are determined to protect implantable and patient-carried parts of an AIMD from the foreseeable electromagnetic environment derived from the European Recommendation 1999/519/EC [11], which was based on the recommendations for General Public of the ICNIRP (International Commission on Non-Ionizing Radiation Protection) Guidelines 1998 [12]. Thus, the risks for a worker who wears an AIMD could be considered acceptable if he/she is exposed to EMF levels below the ICNIRP reference levels for the General Public. In workplaces, powerful sources of EMF are likely to be encountered and reference levels for the General Public can be exceeded. Thus, in workplaces the safety for a worker who wears an AIMD is not guaranteed anymore. In addition, the 45,502 family standards take into account only the EMF sources that can be encountered in common-life scenarios (e.g., GSM/LTE cellular phones, Wi-Fi transmitters). The EMF sources in a work environment can be very specific in terms of modulation, pulse repetition time, etc., and can pose, as a matter of principle, a risk even at levels below the ICNIRP reference levels for the General Public. Consequently, the existing standards reasonably protect the General Public wearing AIMD, but are not sufficient to protect workers wearing AIMD.

For these reasons, the EU has developed a series of technical standards to support the employers in the risk assessment of workers who wear AIMD: the general standard EN50527-1 [13] with the particular standards EN50527-2-X [14.15] for the different AIMD classes.

The EN50527-1 [13] provides a general procedure for the specific assessment required for workers with an AIMD: an initial simplified analysis is required, followed, when necessary, by a deeper specific risk assessment for the AIMD-employee. The initial simplified analysis starts from the identification of all the EMF sources active in the workplace and their comparison with a list of equipment reported in Table 1 (“whitelist”) of the EN50527-1 [13] (Table A.1 of the EN50527-2-1 [14] and of the EN50527-2-2 [15]). Once all the EMF sources have been identified, the simplified analysis can be considered sufficient if:

1. All of the EMF sources are listed in the table;
2. All of the EMF sources are used in accordance with the indication reported in the “exceptions and remarks” column;
3. The AIMD employee has not received specific warnings from the responsible physician that the AIMD may be susceptible to electromagnetic interference (EMI) from one of the present equipment.

If one of the previously mentioned conditions is not verified, a specific risk assessment shall be carried out, in accordance with the specifications provided in Annex A of the standard. The risk assessment should involve input from: (1) The employer and, if applicable, his/her occupational health and safety expert and/or occupational physician; (2) the AIMD employee and his/her responsible physician; and (3) experts (technical and medical), e.g., manufacturer of the AIMD. Then, two alternative methods to perform the risk assessment are proposed: The “non-clinical approach” and the “clinical approach”. The former bases the risk assessment on measurement, calculation, and/or information provided by the manufacturer of the AIMD, and does not involve directly the worker. The latter needs the AIMD employee to be exposed under clinical supervision to the foreseeable exposure situations or in a laboratory simulating the workplace exposure situation. The behavior of the AIMD must then be checked by, e.g., telemetry during and after the exposure. 

The particular standards EN50527-2-1 [14] and EN50527-2-2 [15] follow the same approach as the general standard, providing the procedure for the specific assessment required for workers with implanted PM and ICD, respectively.

In the following part of the paper, three case studies are presented, to better describe how the initial risk assessment can be performed in practice. The general steps adopted are the same for all the three EMF sources considered (RFID readers, Wi-Fi and Bluetooth transmitters, UMTS and LTE phones):

- Step 1: Identification of the exposure scenarios
- Step 2: EMF source characterization:
- Step 3: Literature review
- Step 4: Identification of the applicable technical standards
- Step 5: Specific warnings provided by PM and ICD manufacturers
- Step 6: Risk Assessment

At the end of Step #6, it will be possible to understand if the risk assessment for the specific EMF source can be considered concluded or if a further specific risk assessment analysis is required. 

## 3. Results

### 3.1. Case Report 1: Workers with PM or ICD Exposed to RFID Readers

#### 3.1.1. Identification of the Exposure Scenarios

RFID is an acronym for “radio-frequency identification” and refers to a technology whereby digital data encoded in RFID tags or smart labels (defined below) are captured by a reader via radio waves. 

The RFID readers can be divided into two main categories: (i) readers installed inside gates and (ii) manual hand-held readers. These two categories correspond to two different exposure scenarios:

- The worker that passes through or stops close to the RFID gate;
- The worker that uses or is exposed to a hand-held RFID reader.

#### 3.1.2. EMF Source Characterization

There are several types of RFID tags, some of which are regulated by ISO standards and well-defined operating frequency bands. The characteristics of the systems currently most frequently encountered in the workplace are summarized below:

- 125/134 kHz (LF Low Frequencies, valid worldwide)
- 13.56 MHz (HF High Frequencies, valid worldwide)
- 860–960 MHz (UHF Ultra High Frequencies, depending on the continents they have maximum powers and different frequency bands)

The LF (125/134 kHz) and HF (13.56 MHz) tags are defined by ISO standards as passive tags (without batteries), whereas UHF RFID tags can be active, semi-active or passive. Active tags are powered by batteries, whereas semi-active tags use battery only as the supply for the internal circuitry, while to transmit data to the RFID reader they use part of the energy received from the radio wave generated by the reader itself. Passive tags do not have any internal power source but draw energy from the radio wave sent by the reader that interrogates them, to activate and retransmit the data.

LF and HF technologies are essentially based on the generation of a predominantly magnetic field, while for higher frequencies the electrical component is predominant. Table 1 summarizes the main characteristic of RFID technology in the bands LF, HF and UHF.

In Europe, radio and telecommunication equipment is regulated by the European Directive 2014/53/EU (radio and telecommunication equipment) [16]. The European Telecommunications Standards Institute (ETSI) has developed standards for many short-range devices. The electromagnetic compatibility of RFID systems is regulated by three ETSI standards, which cover the frequency range from 9 kHz to 40 GHz. In particular, the RFID LF and HF systems are regulated by the ETSI EN 300 330-2 standard [17], while the UHF systems from EN 300-220-1 [18] and EN 302–208 [19]. In the United States, RFID systems, as devices that transmit RF energy, are subject to regulation by the Federal Communications Commission (FCC). Table 2 shows the maximum field strengths/transmission powers allowed for RFID systems.

#### 3.1.3. Literature Review

Several papers published in peer-reviewed journals have addressed the compatibility between AIMD and RFID system [20,21,22,23,24]. These studies demonstrate the potential of these technologies to affect the behavior of current PM implanted in patients, at power levels typically adopted by commercial communication devices. LF and HF RFID readers are the most critical sources, in particular when using a pulse generation modality with a repetition time close to the physiological heart rhythm. Examples of interference induced on PM while exposed to RFID readers are: inappropriate pacing inhibition, variation in the programmed pace-to-pace interval, inappropriate pacing (triggering of the asynchronous pacing modality). Mattei et al. [25] showed that the risk assessment of RFID interference against PM/ICD may be difficult due to a misalignment concerning the physical quantities used to express the exposure levels of the RFID and the PM/ICD immunity.

#### 3.1.4. Identification of the Applicable Technical Standards 

PM and ICD, in the European Union, are regulated by new MDR [6], which defines the “essential requirements” they must meet in order to be placed on the market. Immunity to EMFs is an essential requirement for these devices. The European standardization bodies are responsible for developing the corresponding technical specifications that meet the essential requirements of the Directives, the compliance of which will provide a presumption of conformity with the essential requirements. These specifications are called “harmonized standards”. AIMD must comply with the harmonized standard EN 45502-1 [8] and its device-specific standard, which is EN45502-2-1 [9] for PM and EN45502-2-2 [10] for ICD. In the United States, active implantable medical devices must comply with ANSI/AAMI/ISO 14117:2012 Active implantable medical devices—Electromagnetic compatibility—EMC test protocols for implantable cardiac pacemakers, implantable cardioverter defibrillators, and cardiac resynchronization devices [26]. With regard to the electromagnetic compatibility requirements, these standards can be considered substantially equivalent and cover the frequency range 16.6 Hz-3 GHz. 

Although not specifically designed for RFID systems, the following tests cover the PM and ICD immunity in the frequency bands in which the RFID LF, HF and UHF systems operate, according to [9,10]:

- LF—125 kHz and 134 kHz. Several types of tests need to be performed:
  1. Clause 27.3: A continuous sinusoidal signal at various frequencies is applied to the PM/ICD. The amplitude of this signal is 6.25 Vpp (peak-to-peak) at 125 kHz and 6.7 Vpp at 134 kHz. Compliance is confirmed if, once the signal has been applied and then removed, the PM/ICD works as before the test;
  2. Clause 27.4: By applying the same signal, but with an amplitude of 1 Vpp, the PM/ICD must continue to operate without disturbances or in a safe mode defined by the manufacturer even during the application of the interference signal;
  3. Clause 27.5.1: A pulse modulated signal at various frequencies is applied to the PM/ICD. The amplitude of this signal is 0.750 Vpp (peak-to-peak) at 125 kHz and 0.804 Vpp at 134 kHz. Compliance is confirmed if the PM/ICD always works without malfunctions;
  4. Clause 27.8: The PM/ICD is exposed to a magnetic field which varies over time and after the removal of the magnetic field there must be no malfunctions. At the frequency of 125 and 134 kHz, the magnetic field amplitude is 120 A/m and 112 A/m, respectively.
- HF—13.56 MHz. Clause 27.5.3. The test signal is a modulated signal with a carrier frequency of 20 MHz. The signal must be modulated in amplitude to create pulses of 100 ms duration with a peak-to-peak amplitude of 10 V. Compliance is confirmed if the PM/ICD works without malfunctions;
- UHF—865 MHz 915 MHz. Clause 27.5.4 (clause 4.9 of ANSI/AAMI/ISO 14,117 [25]). Radiated tests should be performed using a dipole antenna fed with a pulse modulated signal with a net RF power of 120 mW (RMS). An additional 8 W (RMS) test can be performed voluntarily. The PM/ICD must not exhibit any deviation from the expected behavior during exposure to the RF field.

Notably, concerning the field created by the RFID systems operating in the LF and HF bands, the comparison between the RFID and PM/ICD standards reveals a mismatch in the physical quantities used to express RFID exposure levels and PM/ICD immunity [25]. In RFID regulations, the power limit is expressed in terms of the maximum magnetic field generated by the antenna at a distance of 10 m (in Ampere/meter), while in the PM/ICD standard, immunity is evaluated as the amplitude of a voltage signal directly applied to the input of the device. Since the relationship between the magnetic field at 10 m from the antenna and the voltage induced on the PM/ICD depends on several factors and requires careful electromagnetic modeling, the evaluation of immunity to PM/ICD is neither immediate nor generalizable. 

#### 3.1.5. Specific Warnings Provided by PM and ICD Manufacturers

Some AIMD manufactures explicitly provide in the instruction for use of their products’ special warnings regarding the potential interference with RFID readers. Given the misalignment concerning the physical quantities used to express the exposure levels of the RFID and the PM/ICD immunity, specific warnings provided by the manufacturers may differ. 

**Figure 1:**
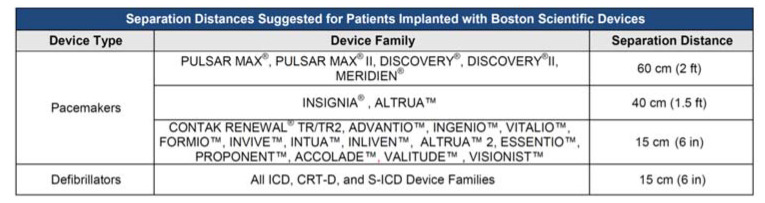
Suggested separation distance between RFID source (transmit antenna) and implanted device, as reported by Boston Scientific in the instruction for use their devices.

Boston Scientific (Marlborough, MA, USA) indicates, for its models, safety distances varying from a minimum of 15 cm to a maximum of 60 cm (Figure 1).

Biotronik (Berlin, Germany), in the technical manuals of its devices indicates that: “Radio-Frequency Identification (RFID)—RFID tags may interact with the CRT-Ps. Patients should be advised to avoid leaving a device containing such a tag within close proximity to the CRT-P (i.e., inside a shirt pocket)”. Notably, the RFID tags mentioned above refer only to active tags, whereas passive tags are not EMF sources and do not pose any EMI risk alone. 

#### 3.1.6. Risk Assessment

RFID systems are not included in the whitelist of the EN50527-1 [13], EN50527-2-1 [14] and EN50527-2-2 [15] standards, therefore in the presence of these sources it is necessary to conduct a specific risk assessment, since the occurrence of malfunction cannot be a priori excluded.

In the LF and HF bands, the analysis of the RFID regulation and the PM/ICD standards reveals a misalignment which makes it difficult to compare RFID exposure levels and PM/ICD immunity. A practical approach is thus to refer to information of use provided by the manufacturers of the AIMD, which are derived from specific not-standardized tests which can differ among manufacturers. 

In the UHF band, the maximum power emitted for RFID readers and PM/ICD immunity are both expressed in terms of watts. However, some considerations are still needed. The 120 mW RF power used in the PM/ICD standards was chosen to ensure compatibility with RF transmitters operating at frequencies close to 900 MHz, with a maximum emitted power of 2 W, at approximately 15 cm. This safety distance could also apply to RFID transmitters operating at similar power (maximum allowed in Europe), but the standard itself recognizes that the specific problem of RFID sources requires further studies and will be specifically addressed in the future versions of the standard. In addition, the 2 W limit is applicable only for the unauthorized use of RFID devices; for specific applications, which may be found in the work environment, it is possible to use a higher level of emitted power when special administrative authorization is obtained.

### 3.2. Case Report 2: Wi-Fi and Bluetooth

#### 3.2.1. Identification of the Exposure Scenarios

Wi-Fi (Wireless Fidelity) technology has achieved significant success and diffusion in many aspects of everyday life and is today the most common communication protocol that allows users to establish and maintain a wireless connection and exchange data via wireless local area networks (WLAN) or Internet. Wi-Fi provides service in homes and private businesses, as well as in public spaces via Wi-Fi hotspots, accessible free of charge or for sale. Wi-Fi sources are therefore to be considered ubiquitous and pervasive. Exposure scenarios for workers with AIMD, such as PM or ICD, are extremely variable and difficult to categorize. Thus, it shall be assumed as worst-case scenario that a worker is constantly exposed to this type of source for a prolonged period.

Bluetooth technology (often abbreviated as BT) is a technical-industrial data transmission standard for wireless personal networks (WPAN). It is used for exchanging data between fixed and mobile devices over short (≈10 m) distances using radio waves. These devices can be, for example, tablets, mobile phones, personal computers, laptops, printers, digital cameras, smartwatches, video game consoles, headphones, provided they are equipped with the hardware and software specifications required by the standard itself. In the last years, BT has also been widely used in the industrial sector (measuring instruments, optical readers, etc.) as the standard communication protocol to exchange data between equipment and data logger, without the need of a wired connection. Like Wi-Fi sources, Bluetooth is a ubiquitous and pervasive source. Exposure scenarios for workers with AIMD, such as PM or ICD, are extremely variable and difficult to categorize. Thus, it shall be assumed as worst-case scenario, that a worker is constantly exposed to this type of source for a prolonged period.

#### 3.2.2. EMF Source Characterization

The IEEE 802.11 standard provides several distinct radio frequency ranges for use in Wi-Fi communications, among which the most commonly used are 2.4 GHz and 5 GHz. In Europe, the highest level of radiated power (in terms of effective isotropic radiated power—EIRP) from Wi-Fi systems depends on the frequency band and the channel within the band itself:

- 2.4 GHz:100 mW (20 dBm)
- 5 GHz channel from 36 to 64:200 mW (23 dBm)
- 5 GHz channel from 100 to 140:1000 mW (30 dBm)
- 5 GHz channel from 155 to 171:4000 mW (36 dBm)

In the United States, Canada and former USSR countries, technical standards grant a higher level of transmitted power (FCC part 15): Wi-Fi devices using an antenna with an isotropic gain of less than 6 dBi cannot exceed an EIRP of 1 W. For antennas with higher directional gain, a transmission up to 4 W is allowed. The 5 GHz channels with powers higher than 200 mW are mainly used for “outdoor” applications, and therefore not in closed environments. Notably, outdoor antennas may be located in places that could be accessible for workers and thus must be carefully considered in the risk assessment analysis.

Regular Bluetooth (BT) operates in the 2.4 to 2.483 GHz industrial, scientific, and medical (ISM) band along with Wi-Fi and lots of other wireless technology. BT devices are divided into three classes, as a function of their transmitting power [27]:

- Class 1: 100 mW ERP
- Class 2: 2.5 mW ERP
- Class 3: 1 mW ERP

The most widely used, Class 2, provides a range up to about 30 m. The higher-power version can reach up to 100 m under the right conditions.

In July 2010, a new version (version 4.0) of the BT standard, called Bluetooth Low Energy (BLE) was released. BLE is designed for ultra-low power consumption, and a typical transceiver is expected to run for years on a single coin cell. It targets ultra-mobile and portable applications in the medical, automotive, consumer wellness, smart energy, entertainment, home automation, security, and sports/fitness markets. BLE is very different from the traditional BT. Significant changes have been made to simplify the design and optimize it for low power consumption, without affecting too much the maximum transmission range (≈50 m). The BLE standard does not define any class based on the transmission power, but provides only the maximum and minimum power values (typical max. power: 10 mW; min. power: 0.01 mW; the maximum output power for low energy devices can reach 100 mW as long as local regulatory bodies allow it [27]).

#### 3.2.3. Literature Review

In a study conducted by Tri et al. [28] at Mayo Clinic College of Medicine (Rochester, MN), six PM and seven ICD were tested in vitro and exposed to the field generated by a Personal Digital Assistant (PDA) connected to a WLAN. No electromagnetic interference has been documented, even when operating in the worst conditions: PM/ICD programmed to the most sensitive setting allowed by the device, Bluetooth transmitting at its maximum power (i.e., 100 mW) and placed near the stimulator.

A more recent study by Mattei et al. [29] did not reveal interference on ten PM exposed to a 2.4 GHz Wi-Fi source with an EIRP up to 20 W and placed in close proximity (about 2 cm) to the PM, therefore under extremely high exposure conditions compared to commercial systems.

The search for published works relating to possible interference between Bluetooth sources and PM/ICD does not lead to any results.

#### 3.2.4. Identification of the Applicable Technical Standards 

At 2.4 GHz, device operation (pacing and sensing) should not be affected when exposed to a modulated signal with a pulse signal activated for 25 ms at 500 ms intervals. The EMF must be generated by a dipole antenna, with a net power of 120 mW RMS (continuous wave).

#### 3.2.5. Specific Warnings Provided by PM and ICD Manufacturers

Boston Scientific (Marlborough, MA, USA) recommends maintaining a distance of 15 cm between the Bluetooth source and the area where the PM or ICD is implanted.

Biotronik (Berlin, Germany) recommends a safety distance of 15 cm for 5 GHz Wi-Fi, and no special precautions for 2.4 GHz Wi-Fi and Bluetooth.

Medtronic (Minneapolis, MN, USA) recommends maintaining a distance of 15 cm between the Bluetooth source and the area where the PM or ICD is implanted. The same distance is suitable for wireless devices for home use. By maintaining this distance, the risk of interference is assumed to be minimal.

#### 3.2.6. Risk Assessment

The analysis of the whitelist of the 50527-2-1 [14] standard (Table A.1 of the standard) allows us to state that Bluetooth devices, if complying with their product standard specifications, do not pose particular risks for workers with a PM. For Wi-Fi systems, there is no risk if the transmission power is less than 120 mW. If the transmission power is greater than 120 mW, a specific risk assessment must be followed. A similar assessment applies to ICD, as described in the EN50527-2-2 [15] standard. Table 3 reports an extract of the whitelist, containing the specific indications for Wi-Fi and Bluetooth transmitters.

### 3.3. Case Report 3: UMTS and LTE

#### 3.3.1. Identification of the Exposure Scenarios

The universal mobile telecommunications system, also known as UMTS (Universal Mobile Telecommunications System), is a 3G cellular mobile phone standard, which is an evolution of the GSM (Global System for Mobile Communications, 2G). Currently, it coexists with the GSM standard, with most mobile phones able to work with both standards based on actual service availability.

Its further evolution is the LTE standard also known as the pre-4G standard. LTE was born as a new generation for broadband wireless access systems and, from a theoretical point of view, is part of the pre-4G segment, placing itself in an intermediate position between 3G technologies such as UMTS and pure fourth generation (4G [LTE Advanced]).

Mobile phones are a ubiquitous and pervasive technology. Exposure scenarios for workers with AIMD such as PM and ICD are the most diverse and difficult to categorize. Thus, it shall be assumed as a worst-case scenario that a worker is constantly exposed to this type of source for a prolonged period.

#### 3.3.2. EMF Source Characterization

The UMTS standard works on 12 frequency bands ranging from 800 MHz to 2700 MHz. The maximum theoretical power emitted by a UMTS phone is 33 dBm (2 W). In Europe the maximum power is limited to 24 dBm (250 mW).

LTE can work on different frequency bands. In particular, the following bands are used in the EU:

- 800 MHz frequency band
- 850 MHz frequency band
- 1800 MHz frequency band
- 1900 MHz frequency band
- 2100 MHz frequency band
- 2600 MHz frequency band

In the EU, the maximum theoretical power emitted by an LTE phone is 23 dBm (200 mW). Table 4 summarizes the main characteristics of mobile telephony and data systems in Europe.

#### 3.3.3. Literature Review

The search on PubMed (https://pubmed.ncbi.nlm.nih.gov, accessed on 30 July 2021) using as keywords UMTS or LTE and PM produced a single result [30]. This in-vivo study in 100 patients with single and dual-chamber pacemakers did not show any malfunctions under worst-case conditions, with the cellular phones positioned directly above the pacemaker pocket.

Searching PubMed with keywords UMTS or LTE and ICD produced a single result [31]. This study, conducted in-vivo on 63 ICD patients, programmed at the maximum sensitivity and with the smartphone placed directly over the ICD casing, did not show any malfunctions.

#### 3.3.4. Identification of the Applicable Technical Standards 

In the 800 MHz to 2.6 GHz band, device operation (pacing and sensing) should not be affected when exposed to a modulated signal with a pulse signal activated for 25 ms at 500 ms intervals. The EMF must be generated by a dipole antenna, with a net power of 120 mW RMS (continuous wave).

#### 3.3.5. Specific Warnings Provided by PM and ICD Manufacturers

All manufacturers recommend a safety distance of the electromagnetic source of at least 15 cm from the implant.

#### 3.3.6. Risk Assessment

Both the EN50527-2-1 [14] and the EN50527-2-2 [15] standards indicate a safety distance of 15 cm, for mobile phone sources up to 2 W of radiated power, as stated in the “whitelist” reported in the standards (Table 5).

## 4. Discussion

Workers who wear AIMD are considered at particular risk if exposed to EMF and, according to the EU Directive 2013/35/EU [2], need an in-depth and individual risk assessment. The EN50527 technical standard family [13,14,15] provides the general procedures that the employer shall follow to carry out the risk assessment. In this paper, three examples of the risk assessment procedure are described. For the three EMF sources taken as examples (RFID readers, Wi-Fi and Bluetooth transmitters, UMTS and LTE phones) a common approach has been identified and is proposed as a possible common approach to be adopted as a general guideline for the risk assessment. The analysis starts from the identification of the possible exposure scenarios, where the ways of interaction between the selected EMF source and the worker with the AIMD have to be defined and characterized in the actual working conditions, in terms of maximum possible distance, maximum allowed power, maximum exposure time, and so on. Then, the EMF source of interest must be properly characterized, in terms of radiated power, operating frequency range, signal modulation and so on. At this stage, the international standard that regulates the emission characteristics of the EMF source must be considered and discussed. The following steps involve: a literature review of the documents (e.g., scientific papers, case reports, technical reports) addressing the compatibility issues between the EMF source and the AIMD of interest; an analysis of the regulatory framework to identify the immunity levels the AIMD shall comply with, in the specific frequency ranges of the EMF source under investigation. The literature review can provide useful information on the likelihood and on the severity of the possible malfunctions caused during the EMF exposure and can also provide valuable data on the possible mechanics of interaction. The identification of the applicable technical standards allows comparing, when possible, the maximum field level generated by the EMF source to the immunity levels that the AIMD shall comply with, according to the international regulation.

An important point that should be considered in the risk assessment procedure is the presence of specific warnings provided by PM and ICD manufacturers in the instruction for use of their devices. Special warnings may arise from different conditions such as a lower immunity with respect to the standard or a particular EMF source not properly covered by the standard. Such information is not only important in the definition of the risk for the worker, but can also help the employee in the development and implementation of the risk mitigation strategies. Another useful piece of information that should be taken into account for a correct risk evaluation is the age of the implanted device (typically several years). Old implants may be immune to EMF levels lower that those today prescribed by international standards, since the standard has been updated from the time of the device implantation. For example, the RF power at which the PM/ICD should be tested in the range 450 MHz–3 GHz changed from 80 mW to 120 mW to account for the new devices (e.g., Wi-Fi transmitters), which transmitted at an EIRP of 100 mW. In addition, for old devices, it may be difficult to retrieve data regarding the type of AIMD implanted as well the mode of its functioning. In such cases, the support of the medical center where the device was implanted becomes crucial for the employee to carry on an adequate risk evaluation.

All the information collected during the steps described above, shall be used as the basis for the final risk assessment, which can result in two possible outcomes:

(1) The analysis and the data produced are sufficient to determine the risk for the worker and to implement the proper risk mitigation strategies;
(2) The occurrence of malfunction in the considered scenarios cannot be excluded and there are not sufficient data to implement proper mitigation strategies. It is thus necessary to further proceed with the analysis and conduct a specific risk assessment.

As for the three case reports presented in this study, the former simplified analysis is suitable for the Wi-Fi, Bluetooth transmitters and UMTS and LTE phones. On the other hand, the RFID readers require a specific risk assessment that shall be carried out in accordance with the specifications provided in the EN50527 family standards. Indeed, RFID systems are not listed in the whitelist, since, although the operating frequencies are considered by the PM/ICD standards, there can be fixed and portable RFID systems that produce field levels not covered by the standardized tests.

An in-depth analysis on how to perform a specific risk assessment goes beyond the aim of this paper. Practical examples of how to perform a specific risk assessment for a worker bearing a PM can be found in [32].

A specific risk assessment shall be performed also in case of a simultaneous exposure to multiple EMF sources. According to the EN 50527-family standards, the assessment of the exposure to EMF must account for all the EMF sources operating in the work environment. If the worker with an AIMD is exposed to multiple EMF sources, but never simultaneously (e.g., because it is activated at a different moment of the work day or because it is installed in different areas), the methodology described in this study can be adopted, considering separately all the EMF sources present on the workplace. On the other hand, if two or more EMF sources determine a simultaneous exposure, the initial simplified analysis based on the comparison with the equipment reported in the whitelists of the standards cannot be adopted anymore. The resulting EMF will be indeed unpredictable and a specific risk assessment becomes necessary.

The analysis of the case reports discussed in this study not only can help in the identification of the steps that shall be adopted for a correct risk assessment, but also provides useful guideline for workers who are going to get an AIMD. A worker who receives an AIMD should be aware of the information necessary to guarantee their safety as soon as they return to the EMF-exposed work environment after implantation. Such information comes from the technical data of the AIMD (e.g., model number, way of functioning, special warnings, etc.) that can be generally found in the user manual of the device, and from the clinical characteristics of the implant (programmed modality, special settings, particular condition of implant, etc.), which are chosen by the physician/medical center responsible for the implant. It is of crucial importance that the worker is prepared to collect all this information and is able to set the evidence necessary for a correct risk evaluation.

## 5. Conclusions

The risk assessment of workers with AIMD that all the employers shall perform to comply with the EU Directive 2013/35/EU involves an in-depth analysis and a precise characterization of the EMF sources present in the workplace. In this paper, we provided practical indications to help the reader in carrying on this task. The general procedure for the risk assessment, performed according to the indication of the EN50527 technical standards family, and the case reports reported in the paper, can be adopted as a general guideline, to be followed to properly perform the initial analysis of the risk assessment of workers with AIMD exposed to EMF sources and to understand if a further specific risk assessment analysis is required or not.

## Figures and Tables

**Table 1:** Main characteristic of RFID technology in the bands LF, HF and UHF.

**Operating Frequency**	LF 125–134 kHz	HF 13.56 MHz	UHF 868–915 MHz
**Maximum Reading Distance**	0.5 m	1–1.5 m	3 m
**Data Transfer Rate**	low	Good	high
**Reading Capability in Presence of Metal Surface or Liquids**	good	Fair	low
**Tag Dimension**	medium/small	medium/small	small
**Specific Standard Defining Transmission Protocol**	no	ISO/IEC 15693 ISO/IEC 14443	no

**Table 2:** Maximum field strengths/transmission powers allowed for RFID systems.

RFID Systems	
Frequency Range	Maximum Field Strength
Low Frequency (LF) 125 and 134 kHz	~64 dBμA/m, at 10 m
High Frequency (HF) 13.56 MHz	42–60 dBµA/m, at 10 m
Ultra High Frequency (UHF) 865–915 MHz	2 W (4 W for 915 MHz in US and Canada only)

**Table 3:** Extract from Table A.1 of the EN50527-2-2 [15] standard, showing the specific indications for Wi-Fi and Bluetooth transmitters.

Designation of Workplace	Examples of Equipment	Exceptions and Remarks
All places	Lighting equipment	Excluding specialized lighting for industrial purposes where the energy is deployed by microwave or radio frequency fields.
All places	Computer and IT equipment not containing wireless communication	No restrictions Hard disks (other than solid state harddiscs) of portable computers and external hard disks should be treated as equipment producing static magnetic fields and be used only with minimum distance of 15 cm between the hard disk and the device.
All places	Computer and IT equipment wireless transmitters communication using Bluetooth Class 1 or WiFi (both typically 100 mW)	lf such equipment contains RFincluding operating at frequencies greater than 385 MHz with peak power radiation greater than 120 mW either follow manufacturer’s recommendations associated with the device restricting their use or perform a special assessment using one of the methods specified in 4.1.2.

**Table 4:** Main characteristics of mobile telephony and data systems in Europe.

Generation	Voice	Data	Carrier Band (MHz)	Pulsing (Hz)	Max. Power (W)
2G	GSM	EDGE	900 1800	2 8 217 1733	2 (900 MHz) 1 (1800 MHz)
3G	UMTS		2100 (900)	100 1500	0.25
4G	VoLTE	LTE	800 1800 2600	1000	0.25

**Table 5:** Extract from Table A.1 of the EN50527-2-1 [14] (upper panel) and EN50527-2-2 [15] (lower panel) standards, showing the specific indications for UMTS and LTE mobile phones.

Designation of Workplace	Examples of Equipment	Exceptions and Remarks
From Table A.1 of the EV50527-2-1		
All places	Mobile phones, smart phones and cordless phones	For pacemakers the interference distance between a GSM phone and pacemaker is 15 cm for radiated peak powers up to 2 W. For DECT phones (250 mW), it is lower.
From Table A.1 of the EV50527-2-2		
All places	Mobile phones, smart phones and cordless phones	For devices the interference distance between a mobile phone and device is 15 cm for radiated peak powers up to 2 W. For DECT phones (250 mW), it is lower.
